# Integrating equity, diversity, and inclusion throughout the lifecycle of artificial intelligence for healthcare: a scoping review

**DOI:** 10.1371/journal.pdig.0000941

**Published:** 2025-07-14

**Authors:** Ting Wang, Elham Emami, Dana Jafarpour, Raymond Tolentino, Genevieve Gore, Samira Abbasgholizadeh Rahimi

**Affiliations:** 1 Department of Family Medicine, McGill University, Montreal, Quebec, Canada; 2 Faculty of Dental Medicine and Oral Health Sciences, McGill University, Montreal, Quebec, Canada; 3 Schulich Library of Physical Science, Life Sciences, and Engineering, McGill University, Montreal, Quebec, Canada; 4 Mila-Quebec AI Institute, Montreal, Quebec, Canada; 5 Lady Davis Institute for Medical Research, Jewish General Hospital, Montreal, Quebec, Canada; National Tsing-Hua University: National Tsing Hua University, TAIWAN

## Abstract

The lack of Equity, Diversity, and Inclusion (EDI) principles in the lifecycle of Artificial Intelligence (AI) technologies in healthcare is a growing concern. Despite its importance, there is still a gap in understanding the initiatives undertaken to address this issue. This review aims to explore what and how EDI principles have been integrated into the design, development, and implementation of AI studies in healthcare. We followed the scoping review framework by Levac et al. and the Joanna Briggs Institute. A comprehensive search was conducted until April 29, 2022, across MEDLINE, Embase, PsycInfo, Scopus, and SCI-EXPANDED. Only research studies in which the integration of EDI in AI was the primary focus were included. Non-research articles were excluded. Two independent reviewers screened the abstracts and full texts, resolving disagreements by consensus or by consulting a third reviewer. To synthesize the findings, we conducted a thematic analysis and used a narrative description. We adhered to the PRISMA-ScR checklist for reporting scoping reviews. The search yielded 10,664 records, with 42 studies included. Most studies were conducted on the American population. Previous research has shown that AI models improve when socio-demographic factors such as gender and race are considered. Despite frameworks for EDI integration, no comprehensive approach systematically applies EDI principles in AI model development. Additionally, the integration of EDI into the AI implementation phase remains under-explored, and the representation of EDI within AI teams has been overlooked. This review reports on what and how EDI principles have been integrated into the design, development, and implementation of AI technologies in healthcare. We used a thorough search strategy and rigorous methodology, though we acknowledge limitations such as language and publication bias. A comprehensive framework is needed to ensure that EDI principles are considered throughout the AI lifecycle. Future research could focus on strategies to reduce algorithmic bias, assess the long-term impact of EDI integration, and explore policy implications to ensure that AI technologies are ethical, responsible, and beneficial for all.

## Author summary

Our research explores the integration of Equity, Diversity, and Inclusion (EDI) principles into Artificial Intelligence (AI) technologies used in healthcare. As AI plays an increasingly important role in healthcare, it is essential that these technologies are developed with EDI in mind to ensure they benefit all populations fairly. However, there is still a lack of comprehensive frameworks that systematically incorporate EDI principles throughout the AI lifecycle. Our review examines existing studies to understand how EDI has been integrated into AI in healthcare. We found that while some AI models have improved by considering factors such as age, race, and gender, there is still little focus on how EDI should be applied during the actual development or implementation of AI in healthcare settings. Additionally, the composition of AI teams, and how they address EDI, has not been sufficiently studied. Based on our findings, we developed a new framework that emphasizes the importance of EDI at every stage of the AI lifecycle. This framework aims to guide researchers and healthcare professionals in creating more equitable and inclusive AI technologies.

## Introduction

Artificial Intelligence (AI), are progressively being utilized across various domains in our current society, including the realm of healthcare [1–3], where they have been used for categorizing diseases [4], screening [5], and diagnoses [6,7]. AI technologies are also employed to enhance health care services, aid the decision-making process [8], and enhance the efficiency of health systems [9]. AI systems could facilitate achieving quintuple aim of care, namely, improved patient experience, better outcomes, lower costs, clinician well-being, and health equity [10,11].

Although AI offers various advantages to healthcare, both AI and health sectors struggle with a deficiency in upholding Equity, Diversity, and Inclusion (EDI) principles and practices [12]. Neglecting EDI factors when implementing AI in healthcare can lead to the potential introduction of bias and discrimination against certain segments of the population [13]. The groups most susceptible to encountering bias and discrimination are typically members of historically and systematically marginalized minorities. [14]. The absence of EDI principles and practices throughout the entire lifecycle of AI technologies in healthcare, encompassing design, development, and implementation, is increasingly recognized in scholarly discussions as a pressing social, ethical, and health-related issue.

While the meanings of Equity, Diversity, and Inclusion are in a state of ongoing development and are likely to continue evolving, it is crucial to acknowledge their contextual nature, which may differ between various settings. EDI principles developed during the American Civil Rights Movement of the 1960s as a reaction to deeply rooted racial discrimination [15]. In healthcare, for example, equity is understood as a multi-faceted idea, involving both the fair treatment of individuals and the recognition of systemic obstacles faced by marginalized groups. [16]. The notion of diversity is also complicated, encompassing the unique experiences and viewpoints of individuals and groups. These experiences are associated with personal characteristics such as personality, as well as identity markers like race, ethnicity, gender, and sexuality, among others. Meanwhile, inclusion pertains to purposeful endeavors aimed at implementing practices that foster a sense of value, support, and respect for the entire community, with particular emphasis on those who are typically underrepresented. In this context, we define EDI in a broader sense as a deliberate endeavor to incorporate a diverse array of social identities and perspectives, including those who are typically marginalized, in decision-making processes concerning social and health matters that have an impact on their lives and well-being.

The existing ethical framework for AI should be broadened to encompass the concept of EDI. Up to this point, research on AI in healthcare has addressed certain ethical considerations. For instance, previous studies have been carried out on ethical fairness, transparency [12,17] and explainability [18,19]. Recent studies [9,20,21] suggest that there is a lack of consideration and integration of EDI principles and practices by studies on the application of AI in healthcare settings. To the best of our knowledge, there are currently no comprehensive review on AI in healthcare that evaluate how studies have considered and integrated EDI concepts, principles, and practices. Therefore, we conducted a comprehensive scoping review to map the literature regarding this gap in knowledge. The objective of this review was to explore what and how EDI principles and practices have been integrated in the design, development, and implementation of AI technologies in healthcare setting. The study allows to identify the current gaps in the integration of EDI in studies on AI in all healthcare settings and to eventually lead to the development of a framework on best practices to integrate EDI concepts, principles, and practices in AI studies for healthcare.

In line with the objectives of this scoping review, the following research questions were developed:

i) To what extent has EDI concepts, principles, and practices been integrated in the lifecycles of AI in healthcare settings?
ii) How have EDI concepts, principles,  and practices been integrated into the design, development, and implementation of AI in healthcare settings?

## Materials and methods

This scoping review was conducted in accordance with the Joanna Briggs Institute guidelines and the updated framework by Levac et al., building on Arksey and O’Malley’s methodology [22,23] and based on our review protocol [24]. We followed six stages: (i) identifying the research question; (ii) identifying relevant studies; (iii) selecting included studies; (iv) charting the data; (v) collating, summarizing, and reporting the results; and (vi) consulting with stakeholders.

### Search strategy

The detailed search strategy was designed with the help of a health sciences librarian. The search was conducted in the following databases: MEDLINE (Ovid), Embase (Ovid), PsycInfo (Ovid), Scopus, SCI-EXPANDED and ESCI (Web of Science Core Collection). The search strategy covered the period from 2005 to April 29, 2022. The starting year, 2005, was chosen because it marks when the World Health Organization emphasized health equity as a cornerstone of the Millennium Development Goals [13]. A preliminary version of the search was conducted using specific MeSH terms and keywords to assure the accuracy and sensitivity of the search to capture the relevant literature. The reference list of all included sources of evidence was screened to identify additional eligible studies. The search terms are available in S1 Appendix.

### Identifying relevant studies and selecting included studies

Following the search, all identified citations were collated and uploaded into EndNote and duplicates removed. The study selection followed a two-step screening process. In the first step, titles and abstracts were screened by two independent reviewers for assessment against the inclusion and exclusion criteria for the review. To qualify for the full-text scan (second step), the title and abstract had to focus on AI and indicators or socio-bio makers of equity or its related concepts (diversity and inclusion) whitin a healthcare setting. Only studies published in English and French languages were included. All research studies irrespective of study design in which the integration of EDI and AI is the primary focus of the publication, were included. Publications including non-research, commentaries, editorials, and individual points of view were excluded.

### Charting the data

Relevant data were extracted from all included studies by two independent reviewers. A draft structured data recording form developed by the reviewers was used and the information was recorded on Microsoft Excel. The draft data extraction form was modified and revised as necessary during the process of extracting data from each included database. Any disagreements that arose between the two reviewers were resolved through discussion with the third reviewer. The data extracted included specific details on the author(s), year of the study, year of the publication, country of the study, method applied in the study, number of participants, objectives of the study, socio-economic variables for measuring equity such as desegregation of data by sex, gender, sexuality, race, ethnicity, economic and social status, type of AI technology and the type of healthcare setting of application and the key findings relevant to the objectives of the scoping review.

### Collating, summarizing and reporting the results

To synthesize the findings, we conducted a thematic analysis and used a narrative description to describe the work according to the study design (quantitative or qualitative), any emerging patterns identified, ethical implications, as well as legal considerations. We reported findings in line with the PRISMA Extension for Scoping Reviews (PRISMA-ScR) [25].

### Consulting with stakeholders

Throughout the review, we kept all study team members informed and regularly sought their feedback on the quality and accuracy of the ongoing data collection. We also shared preliminary findings with a diverse group of stakeholders—spanning disciplines such as engineering, dentistry, medicine, AI systems, AI ethics, communication, and social sciences—including researchers, clinicians, knowledge users, patient representatives, community partners, policymakers, public health agencies, and industry representatives, during a workshop held in Montreal, QC.

## Results

The results are hereby presented under the corresponding objective.

### To what extent has EDI concepts, principles, and practices been integrated in the lifecycles of AI in healthcare settings.

**Fig 1:**
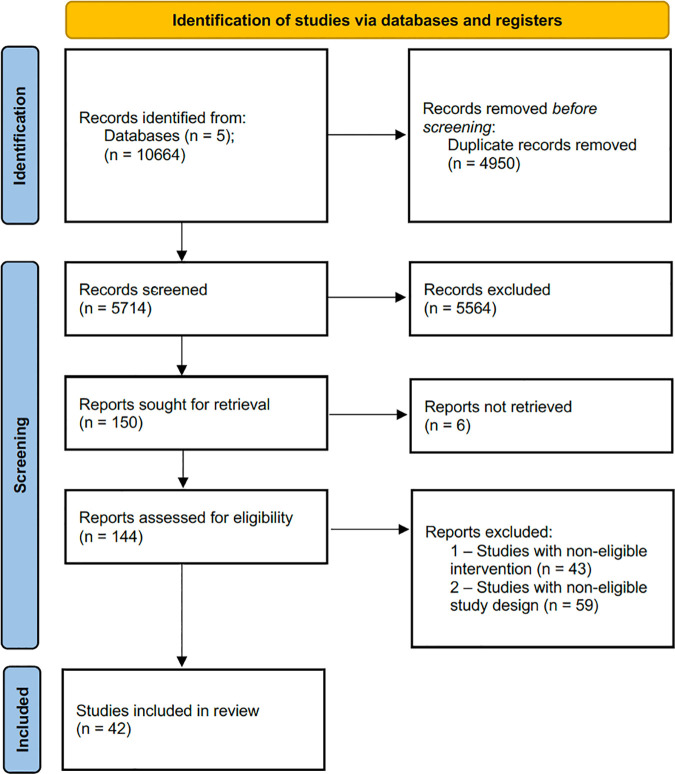
PRISMA flowchart.

The electronic search yielded 10664 records, with 5714 articles remaining after the elimination of duplicates. A total of 150 papers were retained for full text review, and 42 studies were included, as depicted in Fig 1.

**Fig 2:**
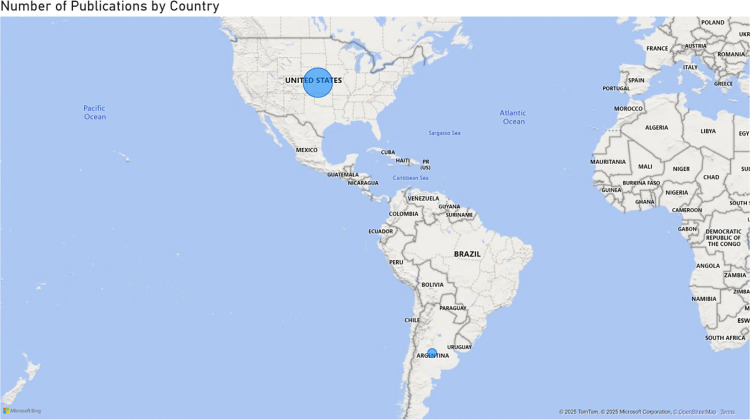
Map of included studies.

Of the included studies, 20 were empirical research, 16 were review papers, and 6 were conceptual narrative research. All the empirical research except for one [26] was conducted on the U.S. population, as depicted in Fig 2.

**Fig 3:**
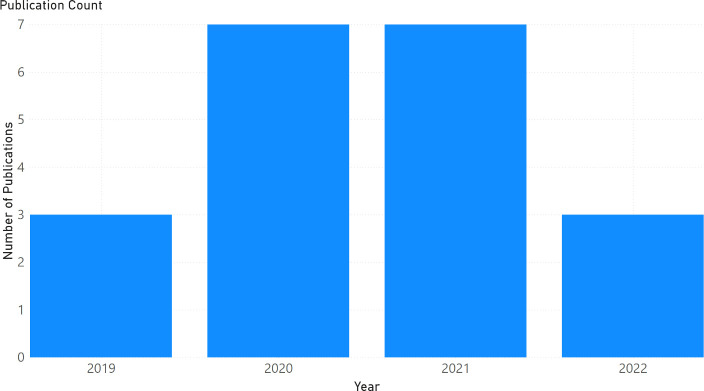
Bar graph of annual publication count.

All included studies were published between 2019 and 2022, as depicted in Fig 3.

The objectives of the included empirical research studies were to detect and evaluate bias in machine learning, develop techniques to mitigate bias, and to use machine learning to detect health disparities. Of the included empirical studies, all were considered as quantitative studies. The sample size in the included studies varied from 234 in the study by Jesse et al. [27] to 1,433,543 in Coley et al. [28]. Regarding the indicators of health equity, of the 20 empirical studies, 8 assessed socioeconomic status (SES), 8 evaluated age, 5 investigated sex, 8 assessed gender, and 16 inspected race/ethnicity.

The sources of data in the included research were electronic health records (EHR) and clinical notes. One of the studies used the public domain Kaggle EyePACS dataset [29], one used National Cancer Database [30], two studied the NIH Chest-XRay14 dataset [14,31], one used cancer omics data, one used Interagency Registry for Mechanically Assisted Circulatory Support [32], and the rest of the studies used EHR from the medical institutions pertaining to each study. The data concerning the AI models and the performance metrics employed in each of the included studies are shown in Table 1.

**Table 1:** Characteristics of data from included empirical studies.

Authors	Objectives	Sample Size	Type of AI	EDI in Design	EDI in Development	EDI in Implementation	EDI in Team
Seyyed-Kalantari et al. [14]	“To examine algorithmic underdiagnosis in chest X-ray pathology classification across three large chest X-ray datasets, as well as one multi-source dataset”	707,626 images from 129,819 patients	Deep learning model, binarized model predictions	Yes	Yes	Not Mentioned (NM)	Not Mentioned (NM)
Fletcher et al. [26]	“To develop a machine learning model for the purpose of decision support, to help general practitioner doctors who do not have significant experience diagnosing pulmonary diseases”	320	A baseline logistic regression model was implemented using the Python Scikit-learn module, with L2 regularization	Yes	Yes	Yes	NM
Ehrenfeld [27]	“To create a natural language processing algorithm to identify transgender patients in electronic health records”	234	Natural language processing algorithm	Yes	Yes	NM	NM
Coley et al. [28]	“To evaluate racial/ethnic differences in the performance of statistical models that predict suicide”	13,980,570 visits by 1,433,543 patients	Logistic regression with LASSO variable selection and random forest	Yes	Yes	NM	NM
Burlina et al. [29]	“To evaluate generative methods to potentially mitigate AI bias when diagnosing diabetic retinopathy resulting from training data imbalance or domain generalization, which occurs when deep learning systems face concepts at test/inference time they were not initially trained on”	88,692 fundi and 44,346 individuals	Deep learning. AI-automated binary diagnostics	Yes	Yes	NM	NM
Nayan et al. [30]	“To evaluate the variation in performance of a machine learning algorithm trained to predict survival after radical prostatectomy in race subgroups”	68,630	Extreme Gradient Boosting classifier, a tree ‐ based, ensemble algorithm	Yes	Yes	NM	NM
Larrazabal et al. [31]	“To provide empirical evidence supported by a large-scale study, based on three deep neural network architectures and two well-known publicly available X-ray image datasets used to diagnose various thoracic diseases under different gender imbalance conditions”	112,120 chest X-ray images from 30,805 patients	Deep neural networks	Yes	Yes	NM	NM
Kostick-Quenet et al. [32]	“To provide an example that illustrates some of the challenges in applying existing guidelines for mitigating algorithmic bias in a ML tool for real-world clinical decision making by physicians and patients. We then discuss the existing legal regulation of AI/ML racial bias and future directions”	25,551	ML algorithm employing Bayesian probability models	Yes	Yes	NM	NM
Chen et al. [33]	“To explore the potential impacts of bias in 2 algorithms, one for predicting patient mortality in an ICU and the other for predicting 30-day psychiatric readmission in an inpatient psychiatric unit. To examine bias, as measured by differences in model error rates in patient outcomes”	3,202	ML topic modeling with latent Dirichlet allocation and logistic regression with L1 regularization	Yes	Yes	NM	NM
Chen et al. [34]	“To determine if it is possible to risk-stratify avoidable utilization without clinical data and with limited patient-level data”	138,115	Decision tree-based machine learning	Yes	Yes	NM	NM
Do et al. [35]	“To propose a joint fairness model approach for logistic regression models for binary outcomes that estimates group-specific classifiers using a joint modeling objective function that incorporates fairness criteria for prediction”	11,594	Logistic regression model	Yes	Yes	Yes	NM
Wylezinski et al. [36]	“To predict Tennessee COVID-19 case growth using machine learning models and investigate the influence of SDOH factors on COVID-19 incidence to quantify and track opportunities to improve health equity”		An ensemble of generalized linear and tree-based machine learning models was built in parallel, each trained and tested with 4–6 weeks of historical COVID- 19 case data to generate predictions from 40 to 50 models at 13 time points	Yes	Yes	NM	NM
Noseworthy et al. [37]	“To determine the impact of race on the performance of CNN to detect the presence of LV systolic dysfunction from an ECG”	97,829	Convolutional neural network	Yes	Yes	NM	NM
Sun et al. [38]	“To analyze EHRs from an urban academic medical center and to investigate whether providers’ use of negative patient descriptors varied by patient race or ethnicity”	18,459	Natural language processing and machine learning, multilevel mixed-effects logistic regression models	Yes	Yes	NM	NM
Allen et al. [39]	“To assess a machine learning algorithm intentionally developed to minimize bias in in-hospital mortality predictions between white and nonwhite patient groups”	53,000	The machine learning mortality predictor was developed using XGBoost, a gradient boosting technique. Gradient boosting combines results from multiple decision trees.	Yes	Yes	NM	NM
Adejare et al. [40]	“To investigate season- and race-specific disparities for asthma risk, and to identify environmental exposure variables associated with ED visits among more than 42,000 individuals of African American and European American descent identified through EHR”	42,375	Machine learning techniques, including random forest, extreme gradient boosting, decision tree, and logistic regression	Yes	Yes	NM	NM
Thompson et al. [41]	“To assess fairness and bias of a previously validated machine learning opioid misuse class”	53,974	Convolutional Neural Network	Yes	NM	NM	NM
Juhn et al. [42]	“To assess the degree to which data quality of EHRs affected by inequities related to low SES, results in differential performance of AI models across SES”	590	2 ML models: Naïve Bayes and gradient boosting machine for binary classification for estimating 1-year AE risk among children with asthma	Yes	Yes	NM	NM
Gao and Cui [43]	“To evaluate and compare extensive set of machine learning experiments on cancer omics data”	447 ML tasks	Deep neural network	Yes	Yes	NM	NM
Obermeyer et al. [44]	“To quantify racial disparities in algorithms and isolate the mechanisms by which they arise”	6,079 patients self-identified as Black and 43,539 patients self- identified as White without another race/ ethnicity	Algorithmic risk score to predict complex health needs	Yes	Yes	NM	NM

### How have EDI concepts, principles, and practices been integrated into the design, development, and implementation of AI in healthcare settings.

EDI integration into the AI lifecycle in healthcare has been explored through empirical research (n = 20), review papers (n = 16), and conceptual narrative research (n = 6). This summary highlights key findings related to three main stages of AI: *design*, *development*, and *implementation*.

#### 1. Design.

By the design stage, we refer to the processes and outcomes of AI analysis. Integration of EDI into this stage has primarily focused on incorporate social determinants of health data in machine learning models [34,36]. The empirical work in this category attempted to detect, measure, and minimize bias in the performance of models predicting health outcomes, by adjusting for the following indicators in their algorithm: age, race, ethnicity, sex, gender, SES (i.e., insurance type, poverty, and income level), biologic markers (i.e., smoking status, BMI, other comorbidities, common chronic disease history, and routinely collected laboratory markers), environmental factors (i.e., firearm crimes, transportation risk, and health infrastructure), health outcomes (i.e., mental health and COVID-19 vaccination), marital status, primary language, COVID-19 test result and encounter, clinical data (i.e., emergency department visits and FEV1%), environmental exposures data (i.e., mold, pollen, and pollutants), and skin color.

Several studies have proposed practical methods for incorporating fairness into AI models. One is example is the work of Do et al. [35], who designed a joint fairness model which incorporated fairness criteria for prediction. The predictive features consisted of demographic variables and biologic markers. Another study by Jesse et al. [27] designed a natural language processing algorithm to pinpoint transgender patients in EHR by using keywords and billing codes.

##### Gaps and recommendations:

Despite some progress, significant gaps remain in the integration of EDI into AI systems. One major concern is the lack of focus on AI ethics in low-to-middle-income settings, alongside the under-representation of diverse patient populations in AI datasets [45]. A series of reviews by Zidaru et al. [46], Daneshjou et al. [47], Jesso et al. [48], Zhao et al. [49], Velagapudi et al. [50], Yi et al. [51], and Straw & Callison-Burch [52] have identified issues such as uneven dataset distribution, under-reporting of race, ethnicity, sex, and socioeconomic factors, and insufficient cross-disciplinary collaboration to address potential biases in AI systems.

To address these challenges, various solutions have been proposed. One approach is Rajkomar et al.’s [53] multi-dimensional fairness framework for machine learning models. It comprises three axes: i) *equal patient outcomes*, meaning that protected patient groups acquire equal profit from the AI; ii) *equal performance*, meaning equal sensitivity, specificity, and positive predictive value, referring to similar accuracy of the model for individuals in the protected and non-protected groups; and iii) *equal allocation*, meaning that the resources are consistently distributed to the protected group. Additionally, experts including Morgenstern et al. [54] advocate for adopting big data standards and algorithms to detect and eliminate societal, cognitive, and selection biases, ultimately promoting fairness.

Other researchers such as Takshi [55] and Grote & Keeling [56] emphasize the need for legislative oversight and ethical frameworks. In their review, Grote and Keeling introduce the *error ratio parity*. This metric acknowledges the complex nature of medical diagnoses, where outcomes may not be uniformly distributed across all groups, and seeks to balance the harms incurred by different groups rather than assuming identical error rates.

Furthermore, Wesson et al. [57] proposed a *sixth V*, *virtuosity* (equity and justice), to the traditional frame of five Vs established for big data (volume, velocity, veracity, variety, and value). They also recommend data augmentation as a means to enhance the representativeness of datasets, thereby promoting greater equity.

Chin and Khozin [58] suggested the creation of a universally accessible “digital highway”— a digital system that provides diverse datasets and AI tools to ensure data equity. The importance of diverse datasets has also been emphasized by other researchers [50,51].

Lastly, Cirillo et al. emphasized the importance of promoting awareness around distinguishing between beneficial and harmful biases in AI [59]. They argue that greater public and scientific awareness is crucial to identifying unintended biases that may emerge in AI systems.

In conclusion, integration of EDI in AI design requires continued efforts to diversify datasets, establish robust ethical frameworks, and promote awareness of potential biases.

#### 2. Development.

Articles were categorized here if they introduced, trained, and tested a new AI model using EDI indicators. Empirical studies demonstrated improved model performance—assessed through metrics such as area under the curve, accuracy, variance, sensitivity, specificity, positive predictive value, and negative predictive value—when adjustments were made based on the following classic sociodemographic EDI indicators:

##### Race/ethnicity:

Chen et al. [33] compared differences in error rates in 30-day psychiatric readmission and ICU mortality for race, gender, and insurance type, and reported that differences between racial groups were not statistically significant, with black patients having the highest error rate for psychiatric readmission. Noseworthy et al. [37] showed consistent performance of a convolutional neural network, which was obtained from a homogeneous cohort of non-Hispanic white population, when detecting low left ventricular ejection fraction across a range of racial/ethnic subgroups in a separate testing cohort. The authors suggested using diverse ethnic, racial, age, and sex groups for any new AI model.

Sun et al.’s [38] model showed that black patients had 2.54 times the odds of having at least one negative descriptor in their notes compared to other ethnicities. Coley et al. [28] developed prediction models for suicide and validated them in a retrospective study. According to the authors, the models accurately predicted the risk of suicide for White, Hispanic, and Asian patients. However, the performance was poor for Black and American Indian/Alaskan Native patients and patients with un-identified race. By preprocessing the training data and ensuring statistical equivalence of false negatives for White and non-White patients, Allen et al. [39] trained their final model as unbiased. Therefore, they were able to develop a model to reduce racial bias.

Adejare et al.’s [40] developed a race-and season-specific predictive classification model. The researchers applied four different machine learning algorithms for building the predictive models from EHR and publicly available environmental exposure data. It was concluded that racial and season-specific disparities exist between asthmatics, which need to be addressed through main predictors such as SES and particulate matter. In Kostick-Quenet et al. [32]’s trained algorithm, no significant role of race as a director predictor of LVAD outcomes were found. Burlina et al. [29] developed a model which showed an accuracy of 73.0% for the baseline diagnostics of lighter-skin individuals versus 60.5% for darker-skins, providing evidence of bias in AI performance across under-represented sub-populations. A machine learning algorithm trained by Nayan et al. [30] showed variation in performance by race, in predicting survival after radical prostatectomy. Obermeyer et al. [44] also showed that Black patients are considerably sicker than White patients. Gao and Cui [43] showed that ethnic composition of the group, omics data type, cancer type, and clinical outcome endpoint, can influence the performance of multi-ethnic machine learning systems (with a lower prediction performance for the disadvantaged group).

##### Other SES factors:

Chen et al. [33] showed that in predicting patient mortality, adding demographic information including patients’ age, race, gender, and insurance type (as a proxy measure of SES), improves the performance of the model and increases the area under the curve. They also showed consistent significant gender differences across data sets, with the highest error rates for female patients for ICU mortality. Kalantari et al. [14] found evidence of underdiagnosis in under-served subpopulations and inter-sectional subgroups (e.g., Black female patients) in the trained algorithm Thus, it is important to consider gender balance in datasets used for AI-based diagnosis in healthcare. Larrazabal et al. [31] also reported a consistent decline in performance of the model for under-represented genders.

Fletcher et al. [26] studied the systematic gender bias by examining the accuracy of the model using equal size homogenous training sets and reporting the performance of the models. For the sampling bias, they developed numerous training sets with varying proportions of each demographic group and then assessed the mean accuracy and variance of the model. According to Chen et al. [34], by using a decision tree-based methods, the researchers were able to develop an AI model which predicts inpatient and emergency department utilization with a high degree of discrimination based on SES features, age, gender, and race.

Do et al. [35] demonstrated the predictive performance of their final model by applying optimal hyper-parameters in the entire training set. They reported better performance of their model across all age groups than the single model. Wylezinski et al. [36] showed that with a rise in vaccination levels, there was a decline in the importance of demographic features such as age, race and ethnicity whereas socioeconomic and environmental risk factors such as poverty, access to transportation and healthcare infrastructure substantially increased. It was, therefore, concluded that measures which promote health equity depend on continuous evaluation of risk mitigation effectiveness. In their developed model, Juhn et al. [42] showed that lower SES is associated with worse predictive model performance. This bias in performance can be due to potential incomplete and inaccurate EHR data.

##### Gaps and recommendations:

In AI development, several ethical considerations and systemic biases were identified as critical challenges. Rajkomar et al. [45] proposed a set of recommendations that span the full lifecycle of AI models, including data collection, training, evaluation, launch review, and monitored deployment. Similarly, Belenguer [60] put forward a structured framework for action, which includes four key stages: testing in a closed environment, testing in an open environment, bias impact assessment, i.e., assessing values such as respect for human autonomy, prevention of harm, fairness, and explicability, and using the model in an open environment, close monitoring and receiving feedback from affected groups.

In addition to these frameworks, Lysaght et al. [61] introduced additional measures that decision-makers can use to guide development. These include core ethical values such as integrity, justice, public benefit, transparency, and accountability. These principles are drawn from the *Ethics Framework for Big Data* [61].

Pot et al. [62] underscored the importance of understanding the social categories that impact data, as well as the quality of datasets used in AI systems. They emphasized that detecting and mitigating bias also requires an understanding of the practices involved in implementing new technologies. While some argue that algorithmic biases are purely technical issues, Pot et al. suggested that these biases can be addressed through improved computational models and more rigorous data collection.

In summary, incorporating EDI into AI development requires comprehensive strategies that involve ethical frameworks, continuous testing, and feedback mechanisms.

#### 3. Implementation.

Few empirical studies have reached this stage among the included works. Do et al. [35] demonstrated that their algorithm is computationally feasible for high-dimensional datasets and applied it in a real-world example to generate fair risk predictions for older, underrepresented COVID-19 patients. Fletcher et al. [26] incorporated the final AI model with EDI principles into an Android mobile application, aimed at aiding general practitioners.

##### Gaps and recommendations:

Reviews often explored this topic in greater detail. Leslie et al. [63] showed that if AI is not implemented in a correct manner, can even exacerbate the disparate effect of COVID-19 on, under-represented, and marginalized populations. Thus, they suggest that mitigating bias requires multi-level interventions across clinical, institutional, societal, and global domains. All stakeholders -decision-makers, technology developers, and health officials must proactively address biases and inequities during the design, development, and deployment of these systems to prevent harm.

The role of education in addressing these challenges is another recurring theme. Zidaru et al. [46] proposed that increasing public understanding of the advantages and shortcomings of AI-assisted healthcare is essential due to ethical and moral concerns about the new technology, as well as the potential risk that AI systems not incorporating EDI could exacerbate social inequalities.

Lysaght et al. [61] build on this by proposing specific ethical principles—such as integrity, justice, transparency, and accountability—that should guide the deployment of AI systems in healthcare.

Zou and Schiebinger [64] highlight the importance of both short-term and long-term strategies to combat systemic biases and health disparities in AI. Short-term actions include diversifying data sources and continuously monitoring AI systems to detect and correct biases. Long-term strategies, require structural changes such as revising funding practices, refining publication standards, and updating educational curricula to include more diverse perspectives on technology and ethics.

Mollura et al. [65] suggest a practical framework for implementing AI in resource-poor communities, which involves a phased approach: building local IT infrastructure, implementing data management regulations, and providing targeted education and training to ensure that AI systems are introduced responsibly and effectively.

**Fig 4:**
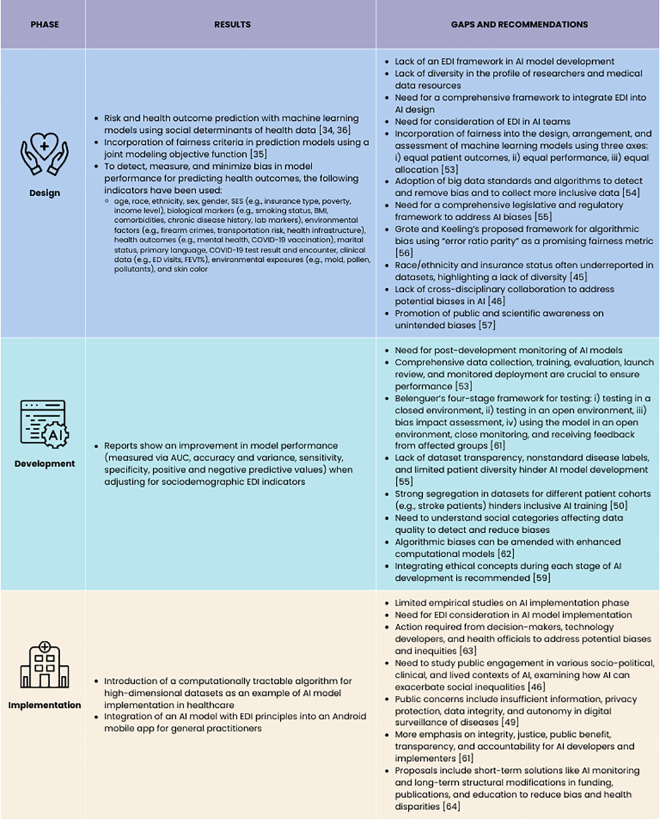
Integration of EDI in design, development, and implementation of the AI models in health sector.

Ultimately, integrating EDI into AI systems' implementation requires a multifaceted approach that includes ethical frameworks, education, strategic policy changes, and continuous monitoring to prevent and mitigate biases, as depicted in Fig 4.

## Discussion

This scoping review was the first to map the current literature with regards to the integration of all EDI principles and concepts into AI lifecycle in healthcare. We found that, currently, the practical integration of EDI into AI by previous studies is rendered through the adjustment of health outcome prediction models by the socio-demographic indicators. Yet not all potential EDI indicators have been adopted by the current algorithms. Besides, while most of the emphasis has been on incorporating EDI into the design and development stages, not much has been done for the implementation phase.

Out of the 20 empirical studies reviewed, 8 examined socioeconomic status, 8 assessed age, 5 focused on sex, 8 investigated gender, and 16 looked at race/ethnicity. However, this is a relatively small number considering there were a total of 42 studies in the review. This suggests that many AI studies in healthcare still do not integrate EDI principles into their design, development, or implementation. As a result, there is a need to ensure that AI systems are applicable to all populations in order to meet the goals of the healthcare quintuple aim.

Regarding reviews on EDI in AI, many identified specific limitations of current AI systems, some in relation to certain diseases (i.e., skin disease), and highlighted how these limitations could worsen existing inequalities. Concerns about responsible AI practices—such as fairness, privacy and security, inclusivity, and transparency—were raised in most reviews. Two of the reviews proposed solutions to address these gaps, focusing on improving representativeness and offering guidelines for decision-makers to ensure that AI systems are developed and implemented in ways that promote equity.

AI systems go through three main lifecycle phases, *design, development*, and *implementation,* whitin an organizational structure, i.e., healthcare setting in the scope of this paper [66]. Therefore, to be able to benefit from a technology and determine its impacts, it is essential to define these pre-requisite stages. According to the definitions provided by Kendall & Kendall [67], the “design” stage refers to “the processes and outcomes of technology analysis and design, including phases such as situational analysis (problems, opportunities, objectives), feasibility analysis, requirements collection, capture, and analysis”. Attempts that have been made by previous studies to integrate EDI to this stage include designing machine learning models to predict health outcomes using social determinants of health data. The indicators that have been used in literature to detect, measure, and minimize bias in model performance for predicting health outcomes have been listed in the paper. However, apart from the classic socio-demographic determinants of health used by previous studies, other EDI indicators such as the educational system, the physical environment, rurality, remoteness and community infrastructure, among many more, can determine health outcomes [68,69]. These indicators of EDI, if not adopted appropriately, can also result in health disparities [24]. Yet, our search did not find any comprehensive framework of EDI indicators to be universally adaptable to any AI model in healthcare. Thus, future studies should consider these indicators by usingframeworks and guidelines which ensures that EDI indicators are properly incorporated in any AI sysetms' lifecycle .

Moreover, a common finding across the included studies was a lack of diversity  whitin the research team. While previous studies have focused on the urge for more diverse data resources [50,51], the importance of diversity in the research team has been mainly disregarded. In this case, we highlight the significance of an inclusive framework which also safeguards the diversity in the profile of the researchers as a prerequisite to the design stage. Inclusion of professionals with diverse backgrounds/characteristics such as gender and racial identities who build up the AI systems can potentially minimize bias, discrimination, and inequitable responses from health services [70]. According to previous studies, a universally accessible digital system, with data augmentation methods can also ensure the inclusion and diversity of data resources at the design stage [58].

The second stage of introducing a new technology is “development”. In the context of AI model development, this stage comprises “the processes and outcomes of defining, testing, coding, training and testing the AI model, the respective data preparation and modeling, and finally preparing for its implementation” [71]. This step is often used interchangeably with the “design” stage, and the conversions are often fluent and delicate. An improvement in the model performance (measured through performance metrics, e.g., the area under the curve, accuracy and variance of the model, sensitivity, specificity, positive predictive value, and negative predictive value) has been reported by the literature standing at this phase of AI model development, when adjusting for the above-mentioned classic sociodemographic EDI indicators. To ensure EDI consideration at this stage, the research team must prioritize transparency, data characterization, inclusive and diverse datasets for AI model training and development. Additionally, they should guarantee constant monitoring of the AI model post-development.

Lastly, the final stage of a technology development is “implementation”. This step refers to “the processes and outcomes of introducing a new technology to the organization, including phases such as user training, documentation, system integration, and data transfer”. Few studies have reached this stage among the included works. Thus, we emphasize the importance of introducing a comprehensive guideline which obliges organizations and funding agencies to ensure that the developed AI technology is equitably implemented in under-represented populations. Future research should also ensure that public education and engagement with the applications and benefits and limitations of AI in health care is also deliberated.

To ensure EDI consideration at all stages, Rahimi et al. recently developed the EDI in AI (EDAI) framework [72]. This framework was created in response to the previously identified gaps, particularly in health and oral health care, and aims to provide a comprehensive guideline for addressing these issues. The EDAI framework was developed in three phases: a scoping review of EDI integration in AI, international workshops with multidisciplinary experts, and iterative refinement based on feedback from these workshops. It offers a structured approach to integrating EDI considerations throughout the AI lifecycle, from data collection to implementation, while addressing individual, organizational, and systemic levels.

### Strengths and limitations

In this scoping review, several notable strengths underscore the rigor and comprehensiveness of the study. Firstly, our comprehensive search strategies ensured that a wide range of relevant literature was identified, minimizing the risk of missing key studies. Additionally, our methodological approaches were conducted meticulously, with careful consideration of study selection and data extraction to maintain the validity of our findings. However, it is essential to acknowledge the limitations of this review. Language restrictions were applied, which could introduce language bias, and the potential for publication bias in the selected studies should be acknowledged as it may affect the overall conclusions. The limitations in this study also included lack of sufficient data for the implementation phase, broad and unclear study design of the included studies, and exclusion of opinion papers. Despite these limitations, the strengths of our review contribute to a robust and insightful overview of the topic.

### Areas for future research

Firstly, we emphasize the need for more studies to apply EDI lenses throughout the design, development, and implementation phases of AI in healthcare. Our review reveals that a limited number of studies have integrated EDI principles and practices into the design, development, and implementation of AI in healthcare settings. Secondly, there should be increased collaboration among research teams to validate our EDAI framework for various use cases and locations. This framework would improve the global implementation of EDI principles and practices, fostering inclusivity in AI-driven decision-making, enhancing trust among diverse populations, and promoting more equitable healthcare outcomes. Thirdly, since very few studies have reached the implementation or deployment stage, future research should prioritize this area as more data becomes available, with an ongoing evaluation of AI’s impact through an EDI lens to ensure ethical, responsible use. Lastly, it is important to explore potential challenges such as resource limitations or resistance to change, and to develop strategies to overcome these barriers as AI technologies continue to evolve.

## Conclusion

AI holds current and future promise for enhancing the delivery and results of healthcare services. However, there persists a deficiency in incorporating EDI principles in the process of designing, developing, and implementing AI in healthcare. This oversight may result in the exclusion of end-users and disadvantaged communities and demographics, who could otherwise experience improved health outcomes through the application of AI technologies in healthcare. Despite a number of frameworks and charters on integration of EDI into AI, there is a lack of a comprehensive framework in healthcare, in which the principles of Equity, Diversity, and Inclusion are systematically deliberated and applied into the AI model development. Herein, we summarized the results of previous studies attempted at integrating EDI principles in the AI model. We have since created a framework in which the principles of EDI are rigorously considered and integrated into the development of the AI model, taking into account all phases of AI lifecycle.

## Supporting information

S1 Checklist PRISMA-ScR checklist. (PDF)

S1 Appendix Search strategy. (DOCX)
