# Supplementary material for: Integrating equity, diversity, and inclusion throughout the lifecycle of artificial intelligence for healthcare: a scoping review
Source: PLOS Digit Health. 2025 Jul 14;4(7):e0000941. doi: 10.1371/journal.pdig.0000941 (PMC12258586; doi:10.1371/journal.pdig.0000941)
Supplement: S1 Appendix — (DOCX) [file pdig.0000941.s002.docx]

**S1 Appendix**

**Search Strategy**

MEDLINE (Ovid)

**2649 records on April 29, 2022**

Ovid MEDLINE(R) ALL <1946 to April 28, 2022>

1 exp artificial intelligence/ or data mining/ or (“artificial intelligence*” or “computational intelligence*” or “machine intelligence*” or “automated reasoning” or “bayesian network*” or “bayes network*” or “naive bayes” or “bayesian learning” or “computer heuristic*” or “computer reasoning” or “data mining” or “text mining” or “expert system*” or “fuzzy logic” or “fuzzy cognitive” or “knowledge representation*” or “knowledge acquisition*” or “machine learning” or “learning machine*” or “natural language processing*” or “neural network*” or “deep learning” or “support vector*” or “hidden markov model*” or “random forest*” or “random decision forest*” or “supervised learning” or “unsupervised learning” or “autoencoder*” or “Generative adversarial network*” or “reservoir computing” or “shallow learning” or “echo state network*” or “case-based reasoning” or “metaheuristic*” or “soft computing” or “approximate reasoning” or “evolutionary computing” or “genetic algorithm*” or “bio-inspired algorithm*” or (competitive learning or learning algorithm* or fuzzy system* or clustering algorithm* or pattern classifi* or document classifi* or fuzzy inference* or fuzzy classifi* or fuzzy rule* or fuzzy control system*)).ti,ab,kf. 281799

2 Health Equity/ 2714

3 gender equity/ or racism/ or sexism/ 8184

4 cultural diversity/ 12544

5 (edi or dei or diversity or inclusion).ti. 77706

6 ((algorithm* adj bias*) or disparit* or equit* or inequit* or inclusivity).mp. 147110

## 7 (diversity and inclusion).mp. 4699

## 8 ((discrimination or discriminatory or diversity or inclusion) and (race or racial or ethnic* or gender* or lgbt* or sex* or social)).mp. 103708

## 9 ageis*.mp. 1946

## 10 racis*.mp. 9775

## 11 sexis*.mp. 4064

## 12 or/2-11 326742

## 13 1 and 12 2779

## 14 limit 13 to yr=“2005 -Current” 2649

## Embase (Ovid)

### **4226 records on April 29, 2022**

Embase <1996 to 2022 Week 16>

1 algorithm/ or algorithm bias/ or exp *artificial intelligence/ or *big data/ or *data mining/ or exp *machine learning/ or exp *robotics/ or *computer vision/ or *expert system/ or *fuzzy logic/ or *natural language processing/ or *genetic algorithm/ or *computer prediction/ or (“artificial intelligence*” or “computational intelligence*” or “machine intelligence*” or “automated reasoning” or “bayesian network*” or “bayes network*” or “naive bayes” or “bayesian learning” or “computer heuristic*” or “computer reasoning” or “data mining” or “text mining” or “expert system*” or “fuzzy logic” or “fuzzy cognitive” or “knowledge representation*” or “knowledge acquisition*” or “machine learning” or “learning machine*” or “natural language processing*” or “neural network*” or “deep learning” or “support vector*” or “hidden markov model*” or “random forest*” or “random decision forest*” or “supervised learning” or “unsupervised learning” or “autoencoder*” or “Generative adversarial network*” or “reservoir computing” or “shallow learning” or “echo state network*” or “case-based reasoning” or “metaheuristic*” or “soft computing” or “approximate reasoning” or “evolutionary computing” or “genetic algorithm*” or “bio-inspired algorithm*” or (competitive learning or learning algorithm* or fuzzy system* or clustering algorithm* or pattern classifi* or document classifi* or fuzzy inference* or fuzzy classifi* or fuzzy rule* or fuzzy control system*)).ti,ab,kf. 569929

2 algorithm bias/ or health equity/ or health disparity/ or health care disparity/ or racial disparity/ 50529

3 gender equity/ or social discrimination/ or ageism/ or exp “discrimination against sexual and gender minorities”/ or racism/ or exp sexism/ 25872

4 cultural diversity/ 2676

5 (edi or dei or diversity or inclusion).ti. 75893

6 ((algorithm* adj bias*) or disparit* or equit* or inequit* or inclusivity).mp. 177044

7 (diversity and inclusion).mp. 5385

8 ((discrimination or discriminatory or diversity or inclusion) and (race or racial or ethnic* or gender* or lgbt* or sex* or social)).mp. 146065

9 ageis*.mp. 2487

10 racis*.mp. 13403

11 sexis*.mp. 4456

12 or/2-11 396833

13 1 and 12 6346

14 limit 13 to yr=“2005 -Current” 6074

15 limit 14 to conference abstract status 1848

16 14 not 15 4226

## PsycInfo (Ovid)

### **1091 records on April 29, 2022**

APA PsycInfo <2002 to April Week 4 2022>

1 exp artificial intelligence/ or exp artificial neural networks/ or conversational agents/ or exp expert systems/ or exp neural networks/ or (“artificial intelligence*” or “computational intelligence*” or “machine intelligence*” or “automated reasoning” or “bayesian network*” or “bayes network*” or “naive bayes” or “bayesian learning” or “computer heuristic*” or “computer reasoning” or “data mining” or “text mining” or “expert system*” or “fuzzy logic” or “fuzzy cognitive” or “knowledge representation*” or “machine learning” or “learning machine*” or “natural language processing*” or “neural network*” or “deep learning” or “support vector*” or “hidden markov model*” or “random forest*” or “random decision forest*” or “supervised learning” or “unsupervised learning” or “autoencoder*” or “Generative adversarial network*” or “reservoir computing” or “shallow learning” or “echo state network*” or “case-based reasoning” or “metaheuristic*” or “soft computing” or “approximate reasoning” or “evolutionary computing” or “genetic algorithm*” or “bio-inspired algorithm*” or (competitive learning or learning algorithm* or fuzzy system* or clustering algorithm* or pattern classifi* or document classifi* or fuzzy inference* or fuzzy classifi* or fuzzy rule* or fuzzy control system*)).ti,ab. 72453

2 exp equity/ or exp diversity/ or fairness/ or exp inclusion/ or exp social equality/ 44884

3 racism/ or antiracism/ or antisemitism/ or implicit bias/ or exp prejudice/ or exp “race and ethnic discrimination”/ or racial bias/ or racial disparities/ or racial justice/ or racial privilege/ 19470

4 sexism/ or employment discrimination/ or sex discrimination/ or stereotyped attitudes/ 12630

5 (edi or dei or diversity or inclusion).ti. 12317

6 ((algorithm* adj bias*) or disparit* or equit* or inequit* or inclusivity).mp. 59122

7 (diversity and inclusion).mp. 2886

8 ((discrimination or discriminatory or diversity or inclusion) and (race or racial or ethnic* or gender* or lgbt* or sex* or social)).mp. 77220

9 ageis*.mp. 1974

10 racis*.mp. 15612

11 sexis*.mp. 4872

12 or/2-11 173170

13 1 and 12 1124

14 limit 13 to yr=“2005 -Current” 1091

## Scopus

### 1377 records on April 29, 2022

TITLE-ABS-KEY ( “algorithm* bias*” OR “biased algorithm*” OR “artificial intelligence*” OR “computational intelligence*” OR “machine intelligence*” OR “automated reasoning” OR “bayesian network*” OR “bayes network*” OR “naive bayes” OR “bayesian learning” OR “computer heuristic*” OR “computer reasoning” OR “data mining” OR “text mining” OR “expert system*” OR “fuzzy logic” OR “fuzzy cognitive” OR “knowledge representation*” OR “knowledge acquisition*” OR “machine learning” OR “learning machine*” OR “natural language processing*” OR “neural network*” OR “deep learning” OR “support vector*” OR “hidden markov model*” OR “random forest*” OR “random decision forest*” OR “supervised learning” OR “unsupervised learning” OR “autoencoder*” OR “Generative adversarial network*” OR “reservoir computing” OR “shallow learning” OR “echo state network*” OR “case-based reasoning” OR “metaheuristic*” OR “soft computing” OR “approximate reasoning” OR “evolutionary computing” OR “genetic algorithm*” OR “bio-inspired algorithm*” OR “competitive learning” OR “learning algorithm*” OR “fuzzy system*” OR “clustering algorithm*” OR “pattern classifi*” OR “document classifi*” OR “fuzzy inference*” OR “fuzzy classifi*” OR “fuzzy rule*” OR “fuzzy control system*” ) AND ( TITLE-ABS-KEY ( “algorithm* bias*” OR “biased algorithm*” OR disparity OR equity OR inclusivity OR ( diversity AND inclusion ) OR ( ( discrimination OR discriminatory OR diversity OR inclusion ) AND ( race OR racial OR ethnic* OR gender* OR lgbt* OR sex* OR social ) ) OR racis* OR ageis* OR sexis* ) OR TITLE ( edi OR dei ) ) AND PUBYEAR > 2004 AND ( LIMIT-TO ( SUBJAREA,”MEDI” ) )

## SCI-EXPANDED, ESCI (Web of Science Core Collection)

### **1321 records on April 29, 2022**

(TS=( “algorithm* bias*” OR “biased algorithm*” OR “artificial intelligence*” OR “computational intelligence*” OR “machine intelligence*” OR “automated reasoning” OR “bayesian network*” OR “bayes network*” OR “naive bayes” OR “bayesian learning” OR “computer heuristic*” OR “computer reasoning” OR “data mining” OR “text mining” OR “expert system*” OR “fuzzy logic” OR “fuzzy cognitive” OR “knowledge representation*” OR “knowledge acquisition*” OR “machine learning” OR “learning machine*” OR “natural language processing*” OR “neural network*” OR “deep learning” OR “support vector*” OR “hidden markov model*” OR “random forest*” OR “random decision forest*” OR “supervised learning” OR “unsupervised learning” OR “autoencoder*” OR “Generative adversarial network*” OR “reservoir computing” OR “shallow learning” OR “echo state network*” OR “case-based reasoning” OR “metaheuristic*” OR “soft computing” OR “approximate reasoning” OR “evolutionary computing” OR “genetic algorithm*” OR “bio-inspired algorithm*” OR “competitive learning” OR “learning algorithm*” OR “fuzzy system*” OR “clustering algorithm*” OR “pattern classifi*” OR “document classifi*” OR “fuzzy inference*” OR “fuzzy classifi*” OR “fuzzy rule*” OR “fuzzy control system*” ) AND ( TS= ( “algorithm* bias*” OR “biased algorithm*” OR disparity OR equity OR inclusivity OR ( diversity AND inclusion ) OR ( ( discrimination OR diversity OR inclusion ) AND ( race OR racial OR ethnic* OR gender* OR lgbt* OR sex* OR social ) ) OR racis* OR ageis* OR sexis* ) OR TI= ( edi OR dei ) ) ) AND (EDN==(“WOS.SCI” OR “WOS.ESCI”) AND SJ==(“NEUROSCIENCES NEUROLOGY” OR “HEALTH CARE SCIENCES SERVICES” OR “MEDICAL INFORMATICS” OR “PUBLIC ENVIRONMENTAL OCCUPATIONAL HEALTH” OR “GENERAL INTERNAL MEDICINE” OR “RADIOLOGY NUCLEAR MEDICINE MEDICAL IMAGING” OR “PSYCHOLOGY” OR “ONCOLOGY” OR “BIOCHEMISTRY MOLECULAR BIOLOGY” OR “PSYCHIATRY” OR “SURGERY” OR “CARDIOVASCULAR SYSTEM CARDIOLOGY” OR “OPHTHALMOLOGY” OR “LIFE SCIENCES BIOMEDICINE OTHER TOPICS” OR “BEHAVIORAL SCIENCES” OR “BIOTECHNOLOGY APPLIED MICROBIOLOGY” OR “RESEARCH EXPERIMENTAL MEDICINE” OR “RHEUMATOLOGY” OR “PARASITOLOGY” OR “SOCIOLOGY” OR “DENTISTRY ORAL SURGERY MEDICINE” OR “VIROLOGY” OR “MEDICAL ETHICS” OR “OBSTETRICS GYNECOLOGY” OR “NURSING” OR “DERMATOLOGY” OR “NUTRITION DIETETICS” OR “GASTROENTEROLOGY HEPATOLOGY” OR “BIOMEDICAL SOCIAL SCIENCES” OR “RESPIRATORY SYSTEM” OR “PEDIATRICS” OR “REHABILITATION” OR “INFECTIOUS DISEASES” OR “ENDOCRINOLOGY METABOLISM” OR “UROLOGY NEPHROLOGY” OR “GERIATRICS GERONTOLOGY” OR “MICROBIOLOGY” OR “ORTHOPEDICS” OR “PHARMACOLOGY PHARMACY” OR “IMMUNOLOGY” OR “SPECTROSCOPY” OR “TRANSPLANTATION” OR “LEGAL MEDICINE” OR “SOCIAL WORK” OR “TROPICAL MEDICINE” OR “EMERGENCY MEDICINE” OR “HEMATOLOGY” OR “SUBSTANCE ABUSE” OR “AUDIOLOGY SPEECH LANGUAGE PATHOLOGY” OR “PATHOLOGY” OR “OTORHINOLARYNGOLOGY” OR “ALLERGY” OR “MEDICAL LABORATORY TECHNOLOGY” OR “TOXICOLOGY” OR “ANESTHESIOLOGY” OR “ANATOMY MORPHOLOGY” OR “INTEGRATIVE COMPLEMENTARY MEDICINE” OR “WOMEN S STUDIES” OR “PHYSIOLOGY” OR “SOCIAL SCIENCES OTHER TOPICS”))

Total =10664

After duplicate removal = 5714

After first level =150

51 final number after second screening

6 full texts not found
